# Spray-Dried Serum for Inhaled Antiviral Therapy

**DOI:** 10.3390/pharmaceutics17121518

**Published:** 2025-11-26

**Authors:** Saveria Germani, Miriam Polichetti, Valentina Garrapa, Giovanna Trevisi, Jonas Füner, Ruggero Bettini

**Affiliations:** 1 Food and Drug Department, University of Parma, Parco Area delle Scienze 27/a, 43124 Parma, Italy; saveria.germani@unipr.it (S.G.);; 2 Preclinics GmbH, Wetzlarer Str. 20, D-14482 Potsdam, Germany; jf@preclinics.com; 3 Preclinics Italia srl, Via N. Sauro 3, 43121 Parma, Italy; vg@preclinics.com; 4 Institute of Materials for Electronics and Magnetism-National Council of Research, IMEM-CNR, Parco Area delle Scienze 37/a, 43124 Parma, Italy; giovanna.trevisi@imem.cnr.it; 5 Interdepartmental Centre for Innovation in Drug Products, Biopharmanet-Tec, University of Parma, Parco Area delle Scienze Pad. 33, 43124 Parma, Italy

**Keywords:** dry powder inhaler, dried immune serum, passive immunotherapy, polyclonal antibodies, SARS-CoV-2

## Abstract

**Background**. Inhalable monoclonal antibodies were explored as therapeutics for respiratory viral infections due to their high specificity, which, however, can become a drawback if virus mutational escape occurs. Serum-derived polyclonal antibodies for prophylaxis reflect the diverse response of the immune system, reducing susceptibility to virus mutations and targeting multiple epitopes. **Objectives**. The aim of this work was the development of inhalable powders containing serum of rats immunized against SARS-CoV-2. **Methods & Results**. In a preliminary screening, combinations of sugar and an amino acid outperformed single excipients in terms of retention of protein size and residual moisture content. Four formulations were further developed on neat and albumin-depleted serum: HPβCD/L-leucine in water, HPβCD/L-leucine in phosphate buffer (KP), trehalose/L-leucine in water and HPβCD/glycine in KP. These were subsequently evaluated for aerosol performance and protein stability. All spray-dried formulations afforded respirable particles (MMAD ≤ 5 µm, FPF 70–80%), with L-leucine reducing hygroscopicity and particle aggregation while improving aerosol dispersibility. **Conclusions**. Albumin did not positively affect aerodynamic properties but provided greater protection of immunoglobulin activity (approximately 80% and 90% in albumin-depleted and neat serum, respectively). Buffer selection had no remarkable impact on the considered parameters. L-leucine with HPβCD offered the best balance of aerodynamic performance and protein stabilization.

## 1. Introduction

Viral respiratory infections, including influenza, respiratory syncytial virus (RSV), rhinoviruses, and more recently, SARS-CoV-2, remain a leading cause of morbidity and, in vulnerable populations, mortality worldwide [1]. These pathogens typically establish infection in the mucosal surfaces of the upper airways, particularly the nasal and bronchial epithelia, which represent the primary sites of viral replication [2,3], and subsequently disseminate to the lungs, causing alveolar damage and eventually systemic spread [4,5]. Although vaccination remains the preferred strategy for reaching long-term immunity and large-scale prevention, passive immunotherapies may be advantageous when immediate protection is required, such as in cases of recent exposure to a virus and during outbreaks of emerging diseases or in pre- and post-exposure prophylaxis. In this context, inhalation therapies that neutralize the pathogen directly at the site of infection are particularly advantageous, as they can reach high concentrations directly in the airways, block early viral replication and limit local damage [6]. Inhalable monoclonal antibodies have been widely explored as therapeutic agents for respiratory viral infections due to their high specificity and potent neutralizing activity against single viral epitopes [7,8]. However, target specificity can become a drawback if virus mutational escape occurs, as in the case of SARS-CoV-2 [9]. Conversely, polyclonal antibodies for prophylactic therapy are typically derived from the serum of immunized animals (e.g., horses or sheep), immunized humans or convalescent patients, and they thus reflect the long-lasting and diverse response generated by the immune system; hence, beside the better cost-effectiveness, they are less susceptible to viruses mutations, as they target multiple epitopes and contain more than one type of neutralizing antibodies [10].

Polyclonal antibodies currently on the market have been primarily limited to the intravenous (IV) or intramuscular (IM) route [10]; nevertheless, evidence is also emerging in support of inhaled delivery. Preclinical studies demonstrated that hyper-enriched anti-RSV IgG administered intranasally effectively prevented viral replication in both upper and lower respiratory tracts, outperforming the monoclonal antibody Synagis^®^ [11]. Moreover, a recent clinical trial confirmed the safety of intranasal administration of human anti-SARS-CoV-2 IgG1 in healthy volunteers [12], suggesting that mucosal delivery could represent a valuable alternative to systemic administration.

Airways can be targeted by administering antibodies either as liquid aerosols or as powder formulations. The latter pharmaceutic form is preferred for biotherapeutics, as it reduces reliance on cold-chain logistics, exhibits improved chemical stability, and lowers susceptibility to microbial contamination [13,14,15]. In addition, dry powders can be engineered, using techniques such as spray drying, to achieve a desired particle size distribution and aerodynamic performance, facilitating efficient and targeted deposition within specific regions of the respiratory tract and thereby maximizing local efficacy [14,16,17]. A critical challenge in the production of spray-dried biopharmaceuticals arises from the fact that the drying process exposes proteins to multiple stresses (e.g., shear and thermal stress), and denaturation can occur at the air–water interface during dehydration; therefore, excipients must be carefully selected to both preserve protein functionality and achieve favorable aerodynamic properties.

Saccharides are among the most widely used excipients for the spray-drying of therapeutic proteins due to their well-documented ability to stabilize labile biomolecules. Two complementary theories have been proposed to explain their protective mechanism. The vitrification theory is grounded in the fact that amorphous sugars are characterized by a glass transition temperature (Tg) below which they form a rigid amorphous glassy matrix that causes kinetic immobilization of the proteins and limits degradation pathways. Thus, sugars characterized by relatively high Tg, such as trehalose, might be effective stabilizers. In parallel, the water-replacement theory suggests that saccharides can stabilize proteins by substituting for hydrogen bonds normally provided by water in the hydrated state, thereby preserving the proteins’ native conformation under dehydrated conditions [18,19,20]. Moreover, small-sized sugars have been reported to form a higher number of hydrogen bonds with proteins due to their reduced steric hindrance, which allows them to occupy structural cavities and support the folded protein conformation [21]. On the other hand, larger cyclic oligosaccharides, such as cyclodextrins, also exhibit stabilizing potential likely due to their ability to interact with the hydrophobic pockets of proteins [22,23,24]. The second class of excipients commonly used for spray drying consists of amino acids. Amino acids are known to stabilize both liquid and solid protein formulations through mechanisms that are not yet fully elucidated, but that partially overlap with those of saccharides (e.g., formation of hydrogen bonds and amorphous matrixes) [19,25]. Moreover, amino acids with a hydrophobic lateral chain, such as L-leucine, are characterized by mild surfactant properties and can reduce tension at the droplets’ surface during drying and hence on a dry particle surface. L-leucine is largely employed in inhalable powder formulations, as a multipurpose excipient, as it provides technological advantages, such as smaller particle size and better aerosol dispersion, in addition to protein stabilization [26]. Finally, the use of buffers can be beneficial to counteract pH variations that may occur during the drying phases [27].

Previous work demonstrated that human hyperimmune serum can be spray-dried into inhalable powders using excipients such as trehalose, improving yield and preserving neutralizing activity [28].

Building on these findings, the aim of the present study was to investigate the impact of excipient selection on antibody preservation and aerosol performance of spray-dried hyperimmune serum from rats immunized against SARS-CoV-2, which was taken as a model viral infection. The serum was partially purified from albumin by fractional precipitation and partially used as neat to investigate the potential benefits of albumin as an aerosolization enhancer and protein stabilizer [29,30].

The formulation development and optimization were conducted with the aim of ensuring serum protein stability in spray drying. In the initial phase, a combined approach of spray- and freeze-drying was used to identify suitable protein-to-excipient ratios and excipient combinations efficient in preserving proteins during dehydration and thermal stress. The investigation focused on sugars and amino acid-based formulations containing either a single excipient or combinations of excipients mixed at a 1:1 weight ratio. Among the sugars, mannitol was chosen as a representative monosaccharide, trehalose as an oligosaccharide and hydroxypropyl-β-cyclodextrin (HPβCD) as cyclic oligosaccharides. The selected amino acids included glycine, chosen for its polar, relatively hydrophilic nature and small molecular size; L-leucine, a commonly used, nonpolar and hydrophobic amino acid; L-phenylalanine, an aromatic amino acid with slightly higher hydrophobicity than L-leucine; and arginine, a polar amino acid positively charged at neutral pH, whose positive charge may interact by electrostatic or ion–dipole interaction [31], with serum proteins exhibiting an overall negative surface charge. The role of the buffer in protein stabilization was also investigated by preparing formulations in both ultrapure water and a low-salt phosphate buffer (KP).

## 2. Materials and Methods

### 2.1. Anti-SARS-CoV-2 Antiserum, Neat and Purified

The immunization of Wistar rats was performed according to the German Animal Welfare Legislation (Authorization number: 2347-A-13-2-2021, 13 February 2021). The number of animals used (n = 6) corresponded to the minimum required to obtain enough biological material for the purposes of the study, in accordance with the principles of reduction and the ARRIVE guidelines.

Six female Wistar rats were injected intramuscularly in m. gastrocnemius with 1/10 of the human dose of the Comirnaty^®^ vaccine (Pfizer/BioNTech; Berlin, Germany + New York, NY, USA) active against both the Wuhan and the Omicron variants of SARS-CoV-2. The animals received a prime injection and a boost three weeks after the first treatment. Blood for serum preparation was collected at animal sacrifice, 35 days after prime administration. Animal sacrifice was performed under anesthesia induced by inhalation of isoflurane (5% *v*/*v*) by exsanguination; the chest cavity was opened, and the heart was punctured by means of a vacuum blood sampling system (21G BD Vacutainer Safety-Lok; BD, Eysins, Switzerland). Approximately 7 mL of blood per rat was collected in Vacuette tubes (8 mL CAT serum Sep Clot Activator; Greiner Bio-One, Frickenhausen, Germany) and, after 30 min of incubation at room temperature, centrifuged for 5 min at 10,000× *g* for serum separation. Obtained sera were pooled and divided into two parts; one part was used and tested as is, while the other part was purified by fractionated precipitation with ammonium sulphate according to the Thermo Fischer protocol to remove albumin [32]. The total protein content in the purified and non-purified serum was quantified by Bradford assay using a Bio-Rad Protein Assay Kit II (Cat. No. 5000002EDU, Bio-Rad; Hercules, CA, USA) according to the manufacturer’s instructions. A stock solution of BSA was prepared by dissolving a weighed amount in ultrapure water to obtain a stock concentration of 1 mg/mL. A set of standard solutions was prepared by diluting aliquots of the stock solutions to give a concentration range of 0–0.5 mg/mL. To fit the standard curve, serum IgG was diluted 1:100. A 96-well microtitration plate was used, in which the wells were pre-filled with 200 μL of Bradford Reagent Concentrate dye (Sigma Aldrich, St. Louis, MO, USA). Then, following the arrangement of the plate, 10 μL of each sample was added in duplicate. After 5 min of incubation, readout of samples and standards was done at a wavelength of 595 nm with the Spark 10 M microplate reader (Tecan; Männedorf, Switzerland). Both purified and non-purified serum were stored at −20 °C until usage.

### 2.2. Excipients and Buffers

Mannitol (Pearlitol 100 SD, Roquette; Lestrem, France), trehalose (ACEF; Fiorenzuola d’Arda, Italy), (2-Hydroxypropyl)-β-cyclodextrin (HPβCD, CycloLab; Budapest, Hungary), L-arginine (Sigma Aldrich; St. Louis, MO, USA), L-phenylalanine (Sigma Aldrich; St. Louis, MO, USA), glycine (Sigma Aldrich; St. Louis, MO, USA), L-leucine (ACEF; Fiorenzuola d’Arda, Italy), and bovine serum albumin BSA (Sigma Aldrich; St. Louis, MO, USA) were obtained for use. Potassium phosphate buffer (KP, 500 mL) was prepared at concentrations of 25 mM, 50 mM and 100 mM by dissolving 0.789 g, 1.578 g and 3.154 g of potassium phosphate monobasic KH_2_PO_4_ (Carlo Erba; Milan, Italy) and 1.168 g, 2.336 g and 4.672 g of potassium phosphate dibasic K_2_HPO_4_ (Sigma Aldrich; St. Louis, MO, USA), respectively, in ultrapure water. The pH of the solution was measured with the pH meter IncLab^®^ expert Pro-ISM (Mettler Toledo; Gießen, Germany) and, if necessary, adjusted to 7.0 ± 0.2.

### 2.3. Powder Preparation of BSA and Serum by Spray- and Freeze-Drying

#### 2.3.1. Spray-Drying of Neat Serum and Purified Serum in Trehalose Formulations at Increasing Protein-to-Excipient Ratio

For the preparation of the samples to be dried, trehalose at increasing concentrations was dissolved in highly purified water, and the obtained solution was added under gentle stirring to the buffered solution of the sera thawed at room temperature, to obtain a final concentration of the neat serum and purified serum of 1.5 mg/mL (Table 1). The solutions obtained were then immediately dried.

The protein formulations were dried with a Mini spray dryer B-290 (Büchi Labortechnik AG; Flawil, Switzerland) equipped with a titanium nozzle of 0.7 mm diameter, a high-performance cyclone with a small product collection vessel and operating with air flow rate of 600 L/min, aspirator 35 m^3^/h, solution feed rate 1 mL/min and inlet temperature of 120 °C (outlet temperature of 80–85 °C).

#### 2.3.2. Freeze Drying of Bovine Serum Albumin (BSA) in Single-Component and Binary Formulations

The role of excipients and buffers in protecting proteins from thermal and physical stress was studied by freeze-drying in a preliminary explorative subset of experiments. For this purpose, a CHRIST ALPHA 2-4 LSC PLUS freeze-dryer (Martin Christ; Osterode am Harz, Germany) was used. BSA was adopted as a model protein to screen single-component and binary formulations and select the most stabilizing excipient combinations. Sugars (mannitol, trehalose, HPβCD) alone or in binary formulations (1:1 weight ratio) with amino acids such as L-arginine, L-phenylalanine, glycine and L-leucine were used by maintaining a 1:1 weight ratio with BSA. The components were dissolved both in highly purified water and in 25 mM KP. All protein solutions were prepared in 5 mL aliquots at a protein concentration of 3 mg/mL in glass vials. The samples were frozen to a temperature of −80 °C before freeze-drying. For lyophilization cycles, the vials were brought to −20 °C and held at that temperature for 15 min before ramping to the primary drying conditions. The primary drying phase was divided into four different sections: section 1 at −20 °C and 0.1 mbar for 25 h, section 2 at −15 °C and 0.1 mbar for 8 h 25 min, section 3 at 0 °C and 0.1 mbar for 6 h 30 min and section 4 at 0 °C and 0.01 mbar for 1 h before arriving at secondary drying. Then, the secondary drying was carried out at 10 °C and 0.01 mbar for 8 h.

#### 2.3.3. Spray Drying of Neat Serum and Purified Serum in Selected Binary Formulations

For sample preparation, the excipients were dissolved either in highly purified water or in 25 mM KP, maintaining the concentration of the solutions at 20 mg/mL, i.e., 1.5 mg/mL proteins in neat serum and purified serum, and 18.5 mg/mL the total concentration of excipients (ratio proteins/excipients 1:12.3 weight), keeping at 1:1 weight, the ratio between sugar and amino acid. Spray-drying of protein formulations was performed as described above.

### 2.4. Powder Characterization

#### 2.4.1. Dynamic Light Scattering (DLS)

The particles obtained after freeze or spray-drying were dissolved in 50 mM KP at a concentration of 0.5 mg/mL. BSA, rat neat serum and purified serum were also diluted in 50 mM KP at the same concentration. Hydrodynamic particle diameters were determined using DLS Zetasizer Nano ZS (Malvern Instruments; Malvern, UK) equipped with a 633 nm laser, using NIBS detection (173° backscatter) at 25 °C.

The reading was performed by sampling the solutions of interest in polystyrene cuvettes and using the “Proteins” method in the Zetasizer-driving software (Version 7.13), with an equilibration time of 30 s and a working temperature of 25 °C. The solutions were prepared immediately before the analysis began by dissolving a small portion of lyophilized and dried powders in various volumes of 50 mM KP buffer to obtain a protein concentration of 0.75 mg/mL. Three measurements were performed for each sample and considered valid if the intercept of the correlation function was between 0.8 and 1.

#### 2.4.2. Scanning Electron Microscopy (SEM)

A Field-Emission Scanning Electron Microscope (Zeiss Auriga Compact, Carl Zeiss; Oberkochen, Germany) was used to investigate the morphology, shape and surface characteristics of the spray-dried powders. Powders were deposited on aluminium stubs covered with carbon tape, and then the particles in excess were removed with a gentle nitrogen flow. The microscope was operated with an accelerating voltage of 1.0 kV, sufficiently low to allow the imaging of micrometric-sized insulating particles without the need for metallization. Images were taken at four different magnifications between 1000× and 20,000×.

#### 2.4.3. Particle Size Distribution by Laser Diffraction

The particle size distribution of spray-dried powders was measured by laser light diffraction with a Spraytec^®^ (Malvern Instruments Ltd.; Malvern, UK). Samples were prepared by suspending 10 mg of each individual powder in 10 mL of cyclohexane (Carlo Erba Reagent; Val de Reuil, France) containing 0.5% *w*/*v* of Span 85 (Fluka Chemika; Neu-Ulm, Germany). To improve homogeneity, the dispersion was put in an ultrasonic bath (8510, Branson Ultrasonics Corporation; Danbury, CT, USA) for 7 min before particle size distribution measurements. The analysis for each sample was conducted at room temperature, keeping the samples in the mixer under agitation at 2000 rpm with a lens obscuration between 18% and 20%. Data are expressed as the volume diameter of the 10th (Dv10), 50th (Dv50) and 90th (Dv90) percentile of the particle population and as the Span value [(Dv90 − Dv10)/Dv50].

#### 2.4.4. Size Exclusion Chromatography (SEC)

Quantification of immunoglobulins dissolved in water solution was performed using an HPLC Agilent 1200 Series equipped with an SEC column (HPLC bioZen 3 μm dSEC-2, 200 Å, LC Column 300 × 7.8 mm, Phenomenex; Torrance, CA, USA) preceded by a guard column (bioZen d-SEC-2 guard column 3 μm, Phenomenex; Torrance, CA, USA). The mobile phase was composed of 100 mM KP previously filtered with a cellulose acetate membrane filter 0.45 µm (Sartorius; Göttingen, Germany) under vacuum and pumped at a flow rate of 0.8 mL/min; the injection volume was 10 μL, and the detection was performed at 280 nm. Each chromatographic run lasted 16 min. The calibration curve for immunoglobulin quantification was constructed by serial dilutions of non-purified serum, prepared on the same day of analysis. Linearity was achieved in the concentration range 0.031–1 mg/mL. Peaks with retention times between 8.5 and 15 min were integrated. The LOQ was 0.064 mg/mL, and the LOD was 0.019 mg/mL.

#### 2.4.5. Dynamic Angle of Repose

The dynamic angle of repose was determined as an indicator of the spray-dried powders’ flow properties. A transparent glass vial, filled with the powder, was fixed horizontally to the rotating arm of a friability measurement instrument (Erweka GmbH; Langen (Hessen), Germany) that rotated for 30 s at 20 rpm. A video of the bottom of the vial was recorded with an iPhone 11 (Apple; Cupertino, CA, USA) and three frames were extracted and analysed with the software ImageJ 64 (NIH; Bethesda, MD, USA) to measure the angle between the horizontal lane and the powder avalanche line during the rolling stage. The classification adopted to determine the flowability of the powders corresponds to the values of angle of repose provided by the European Pharmacopoeia 11.8, 2.9.36.

#### 2.4.6. Thermogravimetric Analysis (TGA)

The moisture content of the spray and freeze-dried powders was measured through thermogravimetric analysis that was performed with a TGA/DSC 1 Star System (Mettler Toledo Inc.; Columbus, OH, USA) equipped with a Heto HMT 200 CBN 18-50 cryostat (Heto Lab Equipment; Allerød, Denmark) set at 22 °C and driven by a STAR^e^ software Version 11 (Mettler Toledo Inc.; Columbus, OH, USA). The analysis was conducted in an inert atmosphere under continuous nitrogen flow (80 mL/min) in a temperature range between 25 and 150 °C, with a temperature increase of 10 °C/min. The powder was placed in a 40 μL ceramic crucible, and the weighing of the samples was carried out directly by the system. The moisture content (% *w*/*w*) was calculated as the mass loss recorded between 25 °C and 150 °C. This mass loss was converted to a percentage of the initial dry sample mass, under the assumption that the recorded mass loss was solely due to the evaporation of residual free water within the sample.

#### 2.4.7. In Vitro Aerodynamic Performance Assessment

The in vitro aerodynamic assessment was performed using a Next Generation Impactor (NGI, Copley Scientific; Nottingham, UK) using the inhalation device RS01 filled with size 3 V-Caps^®^ (Capsugel^®^, Lonza; Verviers, Belgium). The instrument was set to work at a flow of 60 L/min (TPK Copley Scientific, Nottingham, UK) to obtain a pressure drop of 4 kPa through the inhaler with an aspiration time of 4 s. Due to the low active ingredient concentration, three capsules loaded with 20 ± 0.5 mg of powder each were discharged. All stages were washed with 10 mL of ultrapure water, while the induction port and the rubber device adaptor were washed with 25 mL. Each test was carried out in triplicate. The concentration of protein in each sample was quantified by SEC. The cumulative undersized mass percentage of proteins found at each stage was used to create a mass distribution plot relative to the cut-off diameters of each stage according to the European Pharmacopoeia 11.8, 2.9.18. Specifically, the median mass aerodynamic diameter (MMAD) was calculated from the plot of the cumulative undersize percentage of the collected proteins (in probit scale) against the logarithmic cut-off values of each stage, using Microsoft Excel^®^. The MMAD corresponds to the particle size at the midpoint (50%) of the cumulative distribution curve. The plot also enabled the determination of the geometrical standard deviation (GSD), a parameter that measures in logarithmic scale the dispersion of the particles around the MMAD, indicating the width of the particle distribution. GSD was calculated as the square root of the ratio between the size at 84 and 16% of the cumulative distribution curve. Finally, the fine particle fraction (FPF%) and the respirable fraction (RF%) are determined.

The FPF% and RF% are percentages of the mass of emitted particles with aerodynamic size smaller than 5 µm. The FPF was calculated as a percentage relative to the emitted dose (ED), which is the delivered dose minus the amount collected in the induction port (IP), while the RF% also accounted for the particles deposited in the IP.

#### 2.4.8. Anti-Spike Protein (SARS-CoV-2) Enzyme-Linked Immunosorbent Assay (ELISA)

The residual activity of the spray-dried anti-SARS-CoV-2 immunoglobulin was evaluated by an ELISA assay. Wuhan S-protein (Cat. No. 40589-V08B1, Sino Biological Inc.; Beijing, China) was immobilized at a concentration of 2.5 µg/mL on a high bind, half-area 96-well microplate, clear flat bottom (Cat. No. 3690, Corning; Corning, NY, USA) in a 0.05 M sodium carbonate buffer, pH 9.6 and incubated overnight at 4 °C. In all washing steps, plates were washed four times with TBS buffer added with 0.05% Tween 20 (TBS-T). After coating and washing, the plates were blocked for 90 min at room temperature with a 0.2% I-BLOCK™ (Thermo Fischer Scientific; Waltham, MA, USA) solution in TBS-T to prevent unspecific bonds of the immunoglobulins to the plate. The powders were dissolved in blocking buffer at a protein concentration of 100 µg/mL; seven-fold serial dilutions of the samples in 1:4 steps were performed. As reference samples, non-purified serum and purified serum were prepared following the same dilution scheme. After an additional washing step, all samples were added to the ELISA plate in duplicate and incubated for 1 h at room temperature. For detection, a rabbit anti-rat IgG polyclonal antibody HRP-conjugated (Cat. No. orb216296, Biorbyt Ltd.; Cambridge, UK) diluted 1:2500 in blocking buffer was incubated in the plates for 1 h at room temperature after washing with TBS-T buffer. After a final washing step, the colorimetric reaction was induced by incubating tetramethylbenzidine (TMB one, Kementec; Taastrup, Denmark) for 10 min at room temperature and stopping it by adding a 1 M sulfuric acid solution. Finally, the readout was executed with a plate reader Mithras LB 940 (Berthold Technologies, Bad Wildbad, Germany) at a wavelength of 450 nm with background correction at 620 nm.

### 2.5. Statistical Analysis

Experimental data are expressed as mean ± standard deviation (n = at least 3). Statistical significance was evaluated using One-Way ANOVA with multiple comparisons, with significance level set at a *p*-value ≤ 0.05. Before performing the ANOVA test, the normality of the data distribution was assessed using the Shapiro–Wilk test and by evaluating the residuals on the normal QQ plot. Homogeneity of variances was assessed using Bartlett’s test. If the data were normally distributed and the variances were not significantly different, an ordinary one-way ANOVA followed by Tukey’s multiple comparisons test was applied. Otherwise, a non-parametric one-way ANOVA using the Kruskal–Wallis test for multiple comparisons was performed. Statistical analysis was performed with GraphPad Prism v 10 (GraphPad Software Inc; San Diego, CA, USA).

## 3. Results

### 3.1. Selection of the Protein-to-Excipient Ratio and Formulations for Spray Drying

As the first stage of formulation development, powders of purified and non-purified serum with increasing concentrations of trehalose as a bulking agent were produced by spray drying to select an appropriate protein-to-excipient ratio. Since protein content was assessed by Bradford assay, which does not discriminate between IgG and other blood proteins such as albumin, the protein content value used in the calculation was slightly overestimated. However, this approximation was considered acceptable for the purposes of this part of the work. The spray-dried powders were tested for residual immunoglobulin activity compared to reference samples of purified and non-purified serum (Figure S1). A protein-to-excipient ratio below 1:5.7 (by weight) did not ensure the preservation of antibody activity of anti-Spike protein in purified serum. However, increasing the weight ratio above 1:5.7 did not yield proportional benefits in activity retention. Therefore, a ratio of 1:12.3, i.e., 1.5 mg/mL proteins and 18.5 mg/mL trehalose in the feed solution, was selected as it provides sufficient collection volume post-spray drying without disproportionately skewing the weight balance toward excipients. Interestingly, antibody activity dropped significantly when drying purified serum, suggesting that introducing a second excipient such as amino acids into the formulation might improve activity retention by substituting, at least in part, protein–protein interactions in the absence of albumin.

Thereafter, the focus was shifted to two other simple, yet critical parameters—namely aggregation and residual water content—which are indicative of the protein instability resulting from a drying process. Thus, freeze-drying was adopted in a preliminary screening phase, to simultaneously process numerous powder samples with reduced time and material consumption focusing primarily on the effect of the excipient during the drying process. At this stage, three saccharides (mannitol, trehalose, HPβCD) and four amino acids (L-arginine, glycine, L-leucine, L-phenylalanine) were studied alone or combined in dual-excipient formulations to obtain an initial assessment of their stabilizing capacity toward the model protein BSA [27,33], during dehydration under freeze-drying stress. Approaching the excipient screening with BSA enabled the investigation of their stabilizing properties, while minimizing the use of animal-derived materials in line with the 3R principles (Replacement, Reduction, Refinement).

A protein-to-excipient weight ratio of 1:1 was selected, since in lyophilization, relatively small amounts of excipients are generally sufficient to stabilize the formulation compared to spray drying. Furthermore, it was demonstrated that even minimal amounts of sugars can confer stabilization to proteins [34]. Thus, it was expected that this proportion would be sufficient to highlight differences in the interaction of the excipients with the protein in terms of aggregation (relative intensity of the monomer peak) and residual moisture content. The role of the buffer in protein stabilization was also investigated by preparing formulations in both ultrapure water and KP 25 mM, a low-salt phosphate buffer.

Table 2 reports the size of the monomer peak of the protein with the relevant intensity and the residual percent moisture obtained from the tested freeze-dried (FD) formulations. A residual moisture of less than or equal to 10% *w*/*w* was considered acceptable, as a higher water content would indicate poor drying efficiency (i.e., excessive affinity for water, which is undesirable in view of a different drying process such as spray drying) and could be detrimental to the chemical stability of the product. The second evaluated parameter was the retention of the size and intensity of the monomeric BSA peak in the lyophilized formulations compared to the raw BSA material, which had an original size of 9.42 ± 1.35 nm and a peak intensity of 75 ± 12%. Therefore, the formulations showing the main monomeric peak greater than or equal to 75% were deemed acceptable.

The diameter of the monomeric BSA appears slightly larger than the 6–8 nm range typically reported in the literature [35,36]. However, since DLS provides a hydrodynamic diameter measure, the size also depends on the protein’s hydration shell and on its interaction with the surrounding medium, which can lead to an overestimation of the real dimensions [37]. In addition, formulations 22 FD, 28 FD, 32 FD and 33 FD yielded diameters exceeding 11 nm. These larger values may correspond to a peak arising from a mixture of monomeric and dimeric BSA, which cannot be clearly distinguished due to the resolution limits of the technique. Indeed, it is documented that at room temperature, the monomeric and dimeric fractions coexist [38].

Overall, apart from Formulation 9 FD, dual-excipient formulations of amino acids and sugars proved to be more effective in stabilizing the proteins relative to single-excipient formulation, as also reported by Pan et al. [39].

Formulations 16 FD, 18 FD, 19 FD, 23 FD, 27 FD, 31 FD, 32 FD and 33 FD met both the established criteria and were, therefore, selected for the following steps.

The eight selected excipient combinations were used for the lyophilization of neat serum. The serum-to-excipient ratio was maintained at 1:1 (*w*/*w*), and the powders were characterized by thermogravimetric analysis for residual moisture content and DLS for particle size determination. The results obtained from the lyophilization of the serum (S-FD) are shown in Table 3, with the numbering consistent with the BSA-based FD formulations. All powders were below the threshold of 10% *w*/*w* moisture content.

For the size analysis, the characteristics of the lyophilized serum were compared with those of the starting material.

The reference serum sample showed a main peak of 144.0 ± 33.9 nm, reasonably attributable to the presence of aggregates and a mixture of unresolved proteins and a secondary peak of smaller size and intensity, which can be ascribed to monomeric and dimeric serum proteins. This was expected because the serum contained aggregates caused by freeze–thaw cycles applied during sample preparation [40]. In addition, serum is a complex matrix containing proteins of different sizes, which are difficult to discriminate by a relatively low-resolution technique such as light scattering. Therefore, in a preliminary screening context, DLS was adopted as a rapid and comparative method. To evaluate the impact of lyophilization on serum, the Z-average of each sample was used as a reference parameter. The Z-average is obtained from cumulant analysis of DLS data and represents the intensity-weighted mean hydrodynamic diameter of the particles in a sample. Since it is calculated based on the particle size distribution, where larger particles contribute disproportionately by scattering more light, the Z-average was used as a sensitive indicator of changes in aggregation level. The measurement obtained for neat serum (Z-average = 79.9 ± 1.4 nm) was used as the reference value, and the results obtained after lyophilization of the tested formulations were compared against it. Formulations that demonstrated a Z-average comparable to that of the starting sample with least 75% intensity were selected for further process development. Formulations 18 S-FD, 31 S-FD, 32 S-FD and 33 S-FD met this criterion, whereas formulations 16 S-FD, 19 S-FD, 23 S-FD and 27 S-FD showed a marked increase in Z-average with values in the range of 114.1–141.3 nm, indicating a higher degree of aggregation.

### 3.2. Spray Drying of Neat Serum and Purified Serum in Selected Binary Formulation

The excipient combinations that better performed in the lyophilization step (in bold phase in Table 3) were selected for formulating the neat serum and purified serum to be submitted to spray drying (SD), as shown in Table 4. The process yield was calculated as the percentage ratio between the powder deposited in the collector and the weight of the solutes in the feed solution.

All formulations except number 4, gave rise to a yield greater than or equal to 70%. Thus, the impact of the formulation components on the flowability, size distribution and morphology of the powder particles was evaluated.

#### 3.2.1. Flowability and Relative Moisture Content

The relationship between powder flowability and relative moisture content was explored (Table 5). The moisture content ranged from 2 to 6% *w*/*w* and the maximum evaporation rate was recorded between 55 and 64 °C (Figure S2), indicting the loss of adsorbed or surface-bound water. L-leucine is well known for lowering hygroscopicity of spray-dried powders being exposed at the surface of droplets and crystalizing during the drying phase [41,42]. On the other hand, glycine allows higher water uptake when compared to L-leucine [43]. These differences are reflected in the relative moisture content, which is lower in all formulations containing L-leucine.

The evaluation of powder flowability, based on the angle of repose, showed that overall, powders made with non-purified serum had better flow properties than those containing purified serum. This finding is consistent with the fact that albumin is known to enhance the flowability of dried powders due to its capability of reducing the inter-particle adhesion forces [15]. However, this trend is reversed in powders containing glycine, where formulation 8 SD, exhibits excellent flowability despite the high moisture content of the powder. Although high moisture content is typically associated with reduced powder flowability, we speculate that the excellent flowability observed at the highest moisture content may result from the suppression of electrostatic interactions, which tend to reduce flowability when moisture is below a certain value. This hypothesis is supported by previous studies, showing that the relationship between moisture content and flowability is complex and not always linear, with cases of improved flowability at higher moisture levels or with optimal moisture contents that maximize flowability while poorer properties are observed above or below these values [44,45,46].

#### 3.2.2. Particle Size Distribution (PSD), Aerodynamic Behavior and Morphology of Spray-Dried Formulations

**Figure 1:**
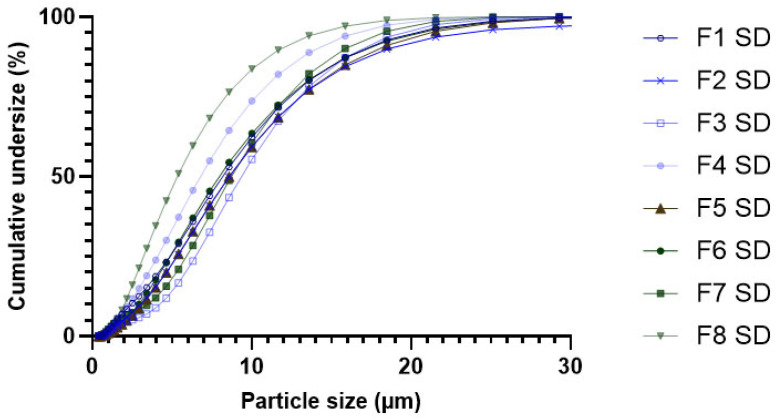
Cumulative undersize percentage distribution of particles volume as a function of the particle size (µm) for formulations 1–8 SD, measured by laser diffraction.

The particle size distribution of the eight spray-dried powders was assessed by laser diffraction and expressed as Dv10, Dv50, Dv90, and Span (Table 6). While some variations in Dv10, Dv50, Dv90, and Span value can be observed, these differences are relatively small and do not suggest remarkably distinct size distribution profiles. The median diameter (Dv50) ranged from 5.33 µm (F8 SD) to 9.34 µm (F3 SD), indicating some variability but generally overlapping size ranges (Figure 1) except for formulation 8, which, curiously, was constituted by the smallest particles despite the excellent flowability.

In general, a distinction can be made between trehalose-containing formulations (F3 SD and F7 SD) and those based on HPβCD. The span values, reflecting the width of the particle size distribution, were lower for the trehalose-containing powders (1.3–1.4) than for HPβCD-containing ones (around 1.8). However, the latter showed a lower Dv10 value, indicating a finer particle fraction. Indeed, trehalose favors the formation of more regular and homogeneous particles, whereas HPβCD generates finer particles, but with a broader size distribution and a greater tendency to aggregate [47].

From the perspective of respirable particle fraction, formulations 4 SD and 8 SD containing HPβCD in combination with glycine appear to be the most promising, as they exhibit a Dv50 slightly above 5 µm. Indeed, the two formulations exhibit a distribution curve shifted toward smaller sizes compared to all other formulations.

All formulations, except for formulations 3 SD and 7 SD, display an asymmetric shape with tailing toward smaller particle sizes (Figure 1). Formulation 2 SD presented a secondary population of particles with diameters around 100 µm, indicating the presence of agglomerates in the powder. Consequently, the undersized distribution curve reaches 100% at larger particle sizes compared to all other powders, due to the contribution of this subpopulation. In contrast, Formulations 3 SD and 7 SD exhibited a more symmetrical main peak, accompanied by a secondary population of particles with diameters around 1 µm. The narrower PSD of formulations 3 SD and 7 SD is consistent with the span values reported in Table 6.

**Figure 2:**
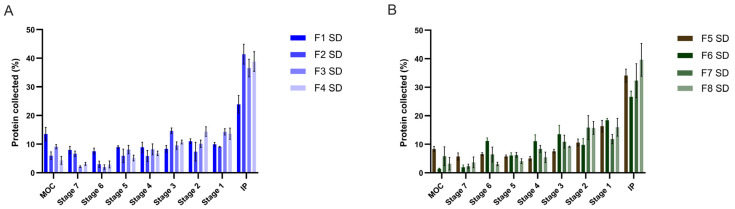
Deposition profiles of spray-dried formulations in the stages of the NGI, in the MOC and in the IP for formulations 1–8. Panel (**A**) shows the deposition profile of formulations 1–4 prepared from non-purified serum, while panel (**B**) of formulations 5–8 containing purified serum. Experiments were conducted in triplicate, and the values are reported as mean ± standard deviation (n = 3).

Subsequently, the aerodynamic properties of the formulations were evaluated via aerosolization studies performed with the NGI. Figure 2 represents the deposition profiles of spray-dried formulations in the stages of the NGI, in the micro-orifice collector (MOC) and in the induction port (IP). For all formulations, the delivered dose was 100%.

The deposition profiles (Figure 2) suggest for all formulations the copresence of smaller and larger particles. As the smaller fraction reaches the deeper stages while the larger fraction is retained in the upper ones, a smoother deposition profile without sharp peaks in specific stages is observed. For all formulations except formulation 1 SD, between 30–40% of the particles were impacted in the induction port, indicating the presence of a coarse, non-respirable particle fraction in the sample.

Respirability parameters, derived from the deposition profiles, are presented in Table 7. Overall, all formulations exhibit a MMAD less than or equal to 5 µm, which ensures good powder respirability and deposition in the deeper regions of the lungs. Indeed, all powders exhibit an FPF% around 70–80% and an RF% ranging from 40 to 60%, which fall within the optimal performance range for inhalation powders [48]. However, significant differences in aerodynamic performance were observed among the formulations.

Focusing on the pairs of formulations prepared with the same excipients (1–5 SD; 2–6 SD; 3–7 SD; 4–8 SD), it is generally observed that neat serum-based formulations containing albumin tend to have lower MMAD values (Table 8). Nonetheless, this trend is reversed in the pair 2–6 SD, and therefore, it is not possible to draw a general conclusion about the influence of albumin on aerodynamic performances. For pairs 1–5 SD and 2–6 SD, the differences in MMAD, FPF and RF% were statistically significant, whereas for pairs 3–7 SD and 4–8 SD, the observed differences were not.

Overall, formulation 1 SD had the best aerodynamic performance, with an MMAD significantly lower than that of the other formulations and the highest FPF% and RF%.

Consistent with its PSD, formulation 2 SD shows the poorest aerodynamic performance, with an MMAD of 5.05 µm, FPF of 69.0% and RF% of 39.3%.

Formulations 4 SD and 8 SD, containing HPβCD and glycine, exhibited MMAD values of 4.09 and 4.90 µm, respectively, and an aerodynamic performance that did not differ significantly from that of formulation 2 SD. This finding contrasts with the PSD measured by laser diffraction, in which the 4–8 SD pair had the smallest Dv50. The discrepancy can be explained by differences in the surface properties conferred by the excipients: while L-leucine migrates to the particle surface during spray drying, forming a hydrophobic layer that reduces interparticle cohesion and enhances dispersibility [41,42], glycine is more zwitterionic and hydrophilic and does not form such an effective surface coating [49]. As a result, when compared to L-leucine, particles containing glycine tend to exhibit stronger cohesion, poorer deagglomeration upon aerosolization and consequently worse aerodynamic performance [50] despite favorable volumetric PSD values. Glycine’s hygroscopic nature further increases residual moisture, which can have a dual effect: while it may act as a lubricant and enhance flowability (see Section 3.2.1), it can also promote liquid bridging between particles, increasing cohesion and ultimately impairing disaggregation and aerosol dispersion.

Finally, GSD values were markedly high for all formulations, indicating a polydisperse distribution, which is typical of therapeutic aerosols [51].

**Figure 3:**
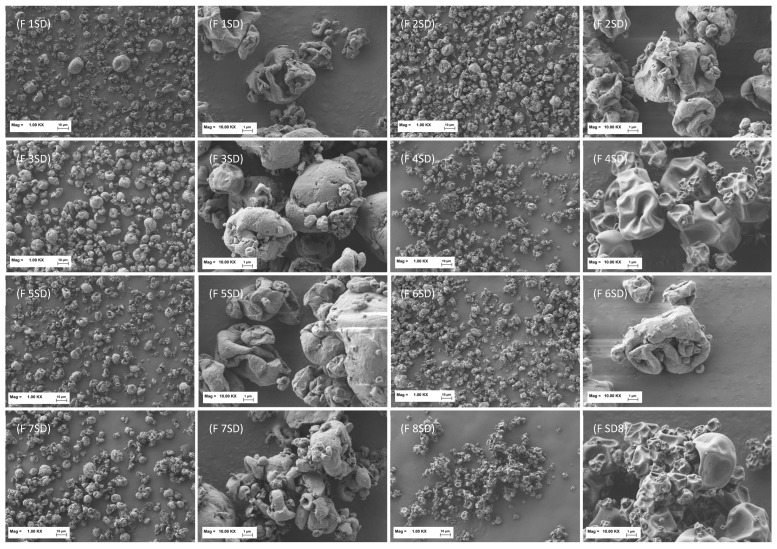
SEM images of spray-dried formulations 1–8 SD (from top to bottom, left to right), each shown at magnifications 1000× (**left**) and 10,000× (**right**).

It is worth underscoring that the formulations tested exhibited good respirability, despite the fact that laser diffraction measurements afforded a Dv50 well above 5 µm for all powders apart from formulation 8 SD. To clarify this aspect, the morphology of the spray-dried particles was investigated by SEM. SEM images at 1000× and 10,000× magnification of the spray-dried formulations are presented in Figure 3.

The prevailing particles morphology is collapsed with fractures at the particles’ surface, suggesting internal hollow cavities. This observation allows us to define the produced spray-dried powders as low-density solids, thus justifying the low MMAD value, which is directly correlated with the morphology and density of the particles. Such properties enhance lung deposition by allowing more efficient transport in the inhaled airstream and reducing inertial impaction in the upper airways [52,53].

In addition, formulations 4 SD and 8 SD containing glycine, presented a smooth surface, while L-leucine-containing particles were corrugated. Surface roughness reduces the actual contact area between particles, thereby decreasing cohesive forces and facilitating deaggregation during aerosolization, so that the release of fine particles is promoted. Moreover, corrugated particles have been reported to display a significantly higher FPF compared with smooth-surfaced particles [54].

Overall, although the glycine-containing particles showed an improved dimensional profile, their reduced surface roughness and higher moisture content—which could promote cohesivity—resulted in poorer aerodynamic performance compared to the other formulations.

#### 3.2.3. Particle Size, Residual Protein Activity and Estimated Pulmonary Activity of Spray-Dried Serum Proteins

Table 9 presents the Z-average values and particle sizes obtained by dynamic light scattering. In both neat serum-based and purified serum-based formulations, the Z-average was consistently lower than in the corresponding reference samples. The raw material had been subjected to an additional freeze–thaw cycle compared to the spray-dried protein formulations. As repeated freeze–thaw cycles can promote aggregation and reduce sample quality, while solid-state formulations may enhance the stability compared to aqueous solutions [55], these factors likely contributed to the observed differences. Formulations containing L-leucine (F1 SD, F2 SD, F3 SD, F5 SD, F6 SD, F7 SD) exhibited a lower Z-average compared to glycine-based formulations (F4 SD, F8 SD) which also showed the highest moisture content (see Section 3.2.1). Residual moisture can enhance the mobility of protein molecules, thus facilitating aggregate formation.

**Figure 4:**
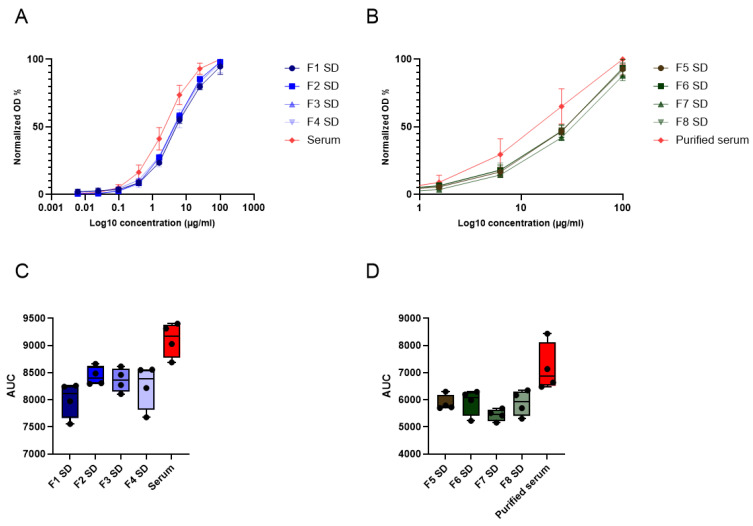
Panels (**A**,**B**): anti-Spike ELISA activity of spray-dried powders and controls (mean ± standard deviation; n = 4). The OD values were normalized to the activity of the reference sample and plotted as a function of logarithmic protein concentration in the range of 0.061–100 μg/mL for neat serum and 0.39–100 μg/mL for purified serum. Panels (**C**,**D**): bar-and-whisker plots (mean values ± standard deviation; n = 4) of AUC values. Statistical significance was assessed by one-way ANOVA followed by Tukey’s multiple comparison test.

To investigate the residual activity of immunoglobulins following the spray-drying process, an anti-Spike protein ELISA was performed, as described in Section 2.4.8. The activity of each powder, measured as optical density (OD) at 450 nm, was normalized to the activity of the reference sample, namely non-purified serum for formulations 1–4 SD and purified serum for formulations 5–8 SD. Normalized OD values were plotted against the total protein concentration in the sample, generating the curves shown in Figure 4A,B. From these curves, the area under the curve (AUC) for each sample was extrapolated using GraphPad Prism v.10 software (Figure 4C,D). For both the powders obtained from neat serum and from purified serum, protein activity in the formulations was lower than that of the relevant reference sample, and consequently, so were the AUCs.

A one-way ANOVA followed by Tukey’s multiple comparison test revealed significant differences between the powders and their respective reference samples (*p* < 0.05 for F2 SD, F3 SD, F5 SD, F6 SD, F8 SD and *p* < 0.01 for F1 SD, F4 SD, F7 SD), but not among the different formulations.

The AUC values of the formulations were normalized to those of the reference samples (9108 ± 322 for neat serum and 7173 ± 888 for purified serum). From the ratio between the AUC of each formulation and its respective reference, the residual protein activity (%) was calculated (Table 10). In addition, the percentage of immunoglobulins able to reach the lower airways was estimated (pulmonary activity %) by correlating residual activity (%) with the FPF (%) obtained in the deposition studies (see Table 7). In general, all spray-dried formulations retained more than 75% of protein activity compared to the reference sample. Protein activity (%) was higher in formulations F1–F4 SD than in F5–F8 SD, confirming the protective role of albumin in preventing denaturation induced by thermal and mechanic stress [56,57], as already observed in preliminary spray-drying studies with trehalose (see Section 3.1).

Consequently, neat serum-based powders exhibited higher estimated lung activity compared to powders formulated with purified serum. Nevertheless, formulations F5–F8 SD also retained more than 50% of lung activity. Among the neat serum-based formulations, F1 SD (L-leucine and HPβCD in water) showed the highest residual lung activity (~67%), together with F3 SD (L-leucine and trehalose in water). For purified serum-based formulations, F5 SD (L-leucine and HPβCD in water) and F6 SD (L-leucine and HPβCD in 25 mM KP) both maintained around 60% pulmonary activity. In contrast, formulations F4–F8 SD showed reduced residual lung activity due to poorer aerodynamic performance, while F7 SD performed worst in terms of protein stability. All tested formulations proved effective in stabilizing proteins during spray drying and in ensuring favorable technological properties of the resulting powders. In detail, L-leucine/HPβCD combinations afforded the most effective formulations for delivering active proteins to the lungs, both for neat and purified serum preparations. Notably, compared to formulations containing only a sugar excipient as the sole excipient, the inclusion of an amino acid led to a marked improvement in protein activity retention, particularly in powders produced with purified serum.

No clear correlation emerged between the aggregation state and the residual protein activity in the formulations. The raw material in liquid form, stored at –20 °C, retained higher activity than the spray-dried formulations, despite showing greater aggregation. Nevertheless, dehydration of serum proteins contributed to overall sample stabilization. Formulations containing L-leucine exhibited a lower degree of aggregation compared to those with glycine, and all formulations were less aggregated than the starting raw material subjected to freeze–thaw. While this reduced aggregation did not translate into a direct benefit in terms of activity retention—likely because it involved various serum proteins rather than specifically the anti-Spike immunoglobulins—the transition to a solid state still represents an advantage. Minimizing aggregation can reduce immunogenic responses and enhance the safety of protein-based therapeutics [58].

## 4. Discussion

The study evaluated strategies to stabilize proteins from purified and neat SARS-CoV-2-immune serum to produce inhalable powders via spray-drying. In the initial phase, a combined approach of spray- and freeze-drying was used to identify suitable protein-to-excipient ratios and excipient combinations efficient in preserving proteins during dehydration and thermal stress. A single-component, trehalose-based formulation was tested at multiple ratios, and 1:12.3 (weight) was selected for spray-drying as it provided a balance between powder yield and activity retention. As some loss of anti-SARS-CoV-2 activity was observed, particularly in purified serum, a broader screening of sugars (mannitol, trehalose, HPβCD) and amino acids (L-arginine, glycine, L-leucine, L-phenylalanine) was performed, studying them alone or combined in dual-excipients formulations both in water and phosphate buffer. The focus then shifted to aggregation and residual water content as key elements of protein instability. Freeze-drying allowed efficient evaluation of excipient effects while minimizing material use. Using BSA as a model protein, excipient combinations that met the selection criteria (≥75% protein size retention; ≤10% *w*/*w* residual moisture) were identified and subsequently tested on serum. The results confirmed that dual-excipient formulations, combining a sugar and an amino acid, provided superior protein stabilization compared with single-component formulations. Four promising formulations (HPβCD/L-leucine in water and phosphate buffer, trehalose/L-leucine in water and HPβCD/glycine in phosphate buffer) were then applied to both purified and neat serum to produce spray-dried powders, which were further characterized for their technological properties, aerosol performance and protein stability in the powders.

Residual moisture (2–6% *w*/*w*) was lower in formulations containing L-leucine, which is less hygroscopic than glycine. Powder flowability, measured by dynamic angle of repose, was superior in neat-serum powders, consistent with the role of albumin in reducing interparticle adhesion. No clear correlation was observed between moisture content and flowability; notably, glycine-based purified serum powders maintained good flowability even at higher moisture, likely due to reduced electrostatic interactions. Particle size distributions showed some variability but generally overlapped (Dv50 5.33–9.34 µm). Trehalose promoted more homogeneous particles (span: 1.3–1.4), while HPβCD generated finer, but more polydisperse powders (span: 1.7–1.8). HPβCD–glycine formulations were the most promising, with Dv50 values near the 5 µm respirability threshold.

Aerosolization studies afforded good respirability for all formulations (MMAD ≤ 5 µm, FPF 70–80%, RF 40–60%) stemming from a hollow structure and low-density particle morphology that favors lung deposition, as revealed by SEM images. L-leucine and glycine confer different surface properties: during spray drying, L-leucine migrates to the particle surface, creating rough, hydrophobic surfaces that reduce cohesion and enhance dispersibility. In contrast, glycine, being relatively hydrophilic, forms smoother surfaces and its higher water content acts as a binder, promoting particle bridging and increasing cohesion. This explains the poorer aerodynamic properties of glycine-containing powders despite favorable particle size distributions.

All proteins retained more than 75% of activity in powder form and showed an estimated pulmonary activity higher than 55%, with neat-serum formulations generally showing higher residual (~90%) and estimated pulmonary activity (~65%), highlighting the protective effect of albumin.

The combination of sugar and amino acid excipients led to a marked improvement in protein activity retention, when compared to the use of a single excipient, proving a higher efficiency of the dual-excipient formulation in replacing water during the dehydration and heating steps of the spray-drying process.

Z-average values determined by dynamic light scattering were lower for all spray-dried formulations when compared to not spray-dried neat and purified serum, with L-leucine-containing powders exhibiting less aggregation than glycine-based ones. Once again, the higher hygroscopicity of glycine is disadvantageous because residual moisture can enhance the mobility of protein molecules, thus facilitating aggregate formation.

No clear correlation was found between aggregation and residual protein activity, as the raw material retained higher activity despite being more aggregated suggesting that aggregation involves various serum proteins rather than specifically anti-Spike immunoglobulins. Nevertheless, dehydration contributed to overall stabilization: all formulations were less aggregated than the raw material after freeze–thaw, highlighting the benefit of drying in reducing aggregation and potentially improving the product safety.

The choice of sugar appears to influence the particle size distribution without affecting aerodynamic properties, with HPβCD-based powders showing finer but less uniform particles.

Buffer type instead affords negligible impact on aerodynamic and protein activity outcomes.

Overall, the amino acid choice was the main factor influencing aerodynamic performance, with L-leucine outperforming glycine in technological properties, as well as in limiting protein aggregation. Albumin clearly improved protein activity retention, but its influence on aerodynamic performances cannot be assessed with certainty.

In conclusion, the four formulations studied were able to effectively preserve proteins during the spray-drying process, while also providing powders with suitable technological properties, ensuring an active pulmonary fraction. Although all four formulations showed good results, the use of L-leucine, particularly in combination with HPβCD, appears to provide the best overall performance in terms of both aerodynamic behavior and protein preservation.

This work addresses formulation issues, while it does not provide evidence of in vivo efficacy, which will be the subject of a subsequent study. Furthermore, with a view to a possible and desirable clinical translation, the importance of GMP-compliant manufacturing, as well as serum compatibility studies, must be emphasized. From this perspective, it is worth noting that the spray-drying technique is widespread and widely available in several FDA or EMA authorized pharmaceutical plants, while the collection and production of plasma, as with all blood products, is subject to rigorous controls and characterizations to ensure not only the absence of potential pathogens but also full compatibility with recipients.

## Figures and Tables

**Table 1:** Trehalose-based formulations of purified and non-purified serum, at increasing protein-to-excipient ratio.

Formulation	Proteins	Trehalose Conc. (mg/mL)	Solid Content Conc. (mg/mL)	Protein/Excipient Ratio
A	Neat serum	3.5	5	1:2.3
B	Neat serum	8.5	10	1:5.7
C	Neat serum	18.5	20	1:12.3
D	Neat serum	28.5	30	1:19
E	Purified serum	3.5	5	1:2.3
F	Purified serum	8.5	10	1:5.7
G	Purified serum	18.5	20	1:12.3
H	Purified serum	28.5	30	1:19

**Table 2:** Size and intensity of the monomer peak along with the residual moisture of single and dual-excipient formulations of freeze-dried BSA. Results are reported as average values ± standard deviation; n = 2 for residue moisture content; n = 6 (two samples analyzed in triplicate) for dynamic light scattering analysis. Residual moisture content (% *w*/*w*) was measured only once for formulations 7 FD, 20 FD and 22 FD due to lack of material.

Formulation	Excipients	Solvent	Moisture Content (% *w*/*w*)	Main Peak Size (nm)	Intensity of Peak (%)
1 FD	Trehalose	KP 25 mM	7.0 ± 3.6	9.33 ± 0.47	52.1 ± 5.3
2 FD	Trehalose	Water	5.2 ± 0.7	9.22 ± 0.64	47.3 ± 3.5
3 FD	Mannitol	KP 25 mM	6.1 ± 1.7	10.04 ± 22.27	51.4 ± 1.6
4 FD	Mannitol	Water	2.9 ± 0.7	8.44 ± 0.52	61.7 ± 4.9
5 FD	HPβCD	KP 25 mM	5.3 ± 0.2	8.70 ± 0.41	52.0 ± 7.5
6 FD	HPβCD	Water	7.4 ± 1.2	9.09 ± 0.26	52.4 ± 4.2
7 FD	Phenylalanine	Water	12.3	8.93 ± 0.72	71.6 ± 14.3
8 FD	L-arginine	KP 25 mM	10.8 ± 2.0	9.11 ± 0.32	55.2 ± 4.6
9 FD	L-arginine	Water	5.3 ± 0.6	8.93 ± 0.34	72.6 ± 10.5
10 FD	L-leucine	KP 25 mM	6.4 ± 2.5	9.45 ± 0.28	55.1 ± 9.7
11 FD	L-leucine	Water	3.1 ± 0.0	8.89 ± 0.29	59.8 ± 6.2
12 FD	Glycine	KP 25 mM	6.4 ± 0.9	8.62 ± 0.86	59.2 ± 10.4
13 FD	Glycine	Water	3.6 ± 0.5	8.82 ± 0.45	55.4 ± 7.0
14 FD	Trehalose/ L-phenylalanine	Water	6.1 ± 0.9	9.93 ± 0.94	48.6 ± 7.1
15 FD	Trehalose/L-arginine	KP 25 mM	22.0 ± 18.8	9.87 ± 2.08	60.5 ± 17.7
16 FD	Trehalose/L-arginine	Water	5.4 ± 0.4	9.67 ± 0.38	80.0 ± 2.6
17 FD	Trehalose/L-leucine	KP 25 mM	5.3 ± 2.8	10.42 ± 1.58	61.9 ± 12.7
18 FD	Trehalose/L-leucine	Water	4.4 ± 0.3	10.29 ± 1.31	86.3 ± 6.2
19 FD	Trehalose/Glycine	KP 25 mM	9.6 ± 2.3	10.48 ± 0.83	77.9 ± 2.6
20 FD	Trehalose/Glycine	Water	7.9	7.97 ± 1.20	41.3 ± 4.3
21 FD	Mannitol/ L-phenylalanine	Water	2.3 ± 0.9	7.46 ± 0.86	55.9 ± 1.4
22 FD	Mannitol/L-arginine	KP 25 mM	13.5	11.85 ± 2.63	15.0 ± 1.8
23 FD	Mannitol/L-arginine	Water	3.3 ± 0.5	8.61 ± 0.61	75.6 ± 1.0
24 FD	Mannitol/L-leucine	KP 25 mM	5.8 ± 3.9	8.16 ± 1.54	31.3 ± 19.7
25 FD	Mannitol/L-leucine	Water	3.9 ± 1.3	8.72 ± 0.14	61.1 ± 2.8
26 FD	Mannitol/Glycine	KP 25 mM	9.1 ± 1.9	9.26 ± 0.93	45.8 ± 13.0
27 FD	Mannitol/Glycine	Water	3.3 ± 1.4	9.18 ± 0.30	76.0 ± 4.3
28 FD	HPβCD/ L-phenylalanine	Water	6.1 ± 0.9	11.84 ± 1.90	64.3 ± 13.3
29 FD	HPβCD/L-arginine	KP 25 mM	6.6 ± 5.2	9.17 ± 0.74	53.3 ± 4.4
30 FD	HPβCD/L-arginine	Water	5.1 ± 1.1	9.33 ± 0.32	55.7 ± 7.4
31 FD	HPβCD/L-leucine	KP 25 mM	5.9 ± 5.0	10.80 ± 0.59	86.1 ± 2.0
32 FD	HPβCD/L-leucine	Water	4.2 ± 1.6	13.11 ± 0.36	86.3 ± 2.8
33 FD	HPβCD/Glycine	KP 25 mM	9.8 ± 0.3	11.70 ± 0.48	82.8 ± 0.6
34 FD	HPβCD/Glycine	Water	7.4 ± 3.0	9.85 ± 0.89	59.4 ± 14.6

**Table 3:** Z-average (d.nm), diameter (nm) and intensity % of both main and secondary peaks of freeze-dried neat serum and neat serum as a reference, along with the relevant residual moisture content (% *w*/*w*). Results are reported as average values ± standard deviation for dynamic light scattering analysis (n = 3). Residual moisture content (% *w*/*w*) was measured once for each formulation.

			Main Peak		Secondary Peak	
Sample	Moisture Content (% *w*/*w*)	Z-Average (d.nm)	Size (nm)	Intensity (%)	Size (nm)	Intensity (%)
Neat serum	-	79.5 ± 1.4	144.0 ± 33.9	90.8 ± 2.4	12.5 ± 0.6	7.1 ± 0.7
16 S-FD	6.76	141.3 ± 0.5	217.1 ± 11.5	96.6 ± 1.5	8.4 ± 0.0	2.0 ± 0.0
18 S-FD	5.25	85.9 ± 0.2	149.7 ± 25.8	92.8 ± 3.0	13.5 ± 1.0	8.0 ± 1.4
19 S-FD	9.10	126.7 ± 3.9	226.9 ± 63.2	85.8 ± 19.7	46.9 ± 52.1	18.9 ± 23.1
23 S-FD	7.51	138.7 ± 6.9	199.2 ± 12.9	95.8 ± 4.4	15.6 ± 0.0	5.6 ± 0.0
27 S-FD	5.41	114.1 ± 3.1	170.0 ± 6.8	95.3 ± 5.1	15.7 ± 16.1	4.7 ± 5.1
31 S-FD	6.67	93.2 ± 2.3	141.7 ± 5.6	93.0 ± 0.7	13.8 ± 1.4	6.0 ± 0.3
32 S-FD	4.95	84.1 ± 3.4	129.8 ± 6.8	92.5 ± 1.9	15.3 ± 2.3	7.2 ± 1.6
33 S-FD	9.59	102.8 ± 1.6	152.6 ± 10.3	94.8 ± 3.4	10.0 ± 8.8	3.6 ± 3.1

**Table 4:** Selected dual-excipient formulations for neat serum, purified serum and spray drying process yield.

Formulation Nr.	Proteins	Excipients	Solvent	% Process Yield
1 SD	Neat serum	HPβCD/L-leucine	Water	77.5
2 SD	Neat serum	HPβCD/L-leucine	KP 25 mM	79.6
3 SD	Neat serum	Trehalose/L-leucine	Water	76.4
4 SD	Neat serum	HPβCD/Glycine	KP 25 mM	43.1
5 SD	Purified serum	HPβCD/L-leucine	Water	78.2
6 SD	Purified serum	HPβCD/L-leucine	KP 25 mM	79.9
7 SD	Purified serum	Trehalose/L-leucine	Water	69.7
8 SD	Purified serum	HPβCD/Glycine	KP 25 mM	81.4

**Table 5:** Flowability obtained by dynamic angle of reposed measurement (mean ± standard deviation, n = 3) and moisture content (% *w*/*w*) of formulations 1–8 SD.

Formulation Nr.	Dynamic Angle of Repose (°)	Flowability	Relative Moisture Content (% *w*/*w*)
1 SD	37.10 ± 0.28	Fair	2.61
2 SD	39.16 ± 0.26	Fair	4.32
3 SD	42.53 ± 0.59	Passable	3.3
4 SD	48.09 ± 0.75	Poor	5.68
5 SD	50.76 ± 0.66	Poor	2.52
6 SD	45.55 ± 0.78	Passable–Poor	3.43
7 SD	51.49 ± 0.49	Poor	2.95
8 SD	28.68 ± 0.40	Excellent	6.29

**Table 6:** Values of Dv10, Dv50, Dv90 in μm and Span for formulations 1–8 SD. The values reported are the average results of 181 technical replications operated by the laser diffractor.

Formulation Nr.	D_V_10	D_V_50	D_V_90	Span
1 SD	2.43	8.16	17.01	1.78
2 SD	3.02	8.58	18.52	1.80
3 SD	4.25	9.34	16.81	1.34
4 SD	2.27	6.78	13.99	1.73
5 SD	3.15	8.59	17.90	1.72
6 SD	2.93	7.96	17.11	1.78
7 SD	3.49	8.71	15.81	1.41
8 SD	2.01	5.33	11.79	1.83

**Table 7:** Respirability parameters of formulations 1–8 SD. MMAD = median mass aerodynamic diameter; GSD = geometric standard deviation, FPF = fine particle fraction; RF = respirable fraction. The values are reported as mean ± standard deviation and 95% confidence intervals of the mean value (n = 3).

Formulation	MMAD (μm)		GSD		FPF%		RF%	
	Mean ± sd	CI 95%	Mean ± sd	CI 95%	Mean ± sd	CI 95%	Mean ± sd	CI 95%
1 SD	1.69 ± 0.29	0.97–2.41	4.46 ± 0.90	2.22–6.70	77.3 ± 0.5	76.1–78.6	58.9 ± 2.6	52.5–65.4
2 SD	5.05 ± 0.39	4.10–6.00	2.89 ± 0.03	2.81–2.96	69.0 ± 0.9	66.7–71.3	39.3 ± 0.1	39.0–39.6
3 SD	3.06 ± 0.12	2.77–3.36	4.40 ± 0.37	3.48–5.32	73.8 ± 1.0	71.5–76.4	48.4 ± 3.1	40.8–56.0
4 SD	4.09 ± 0.50	2.85–5.33	3.61 ± 0.28	2.92–4.23	71.2 ± 1.4	67.7–74.7	43.4 ± 1.9	38.7–48.1
5 SD	2.96 ± 0.54	1.62–4.30	8.83 ± 0.32	8.05–9.63	72.7 ± 1.3	68.9–76.5	47.6 ± 1.6	44.8–50.5
6 SD	3.28 ± 0.16	2.87–3.69	3.66 ± 0.77	1.75–5.56	74.0 ± 1.0	71.5–76.5	54.8 ± 2.0	49.8–59.9
7 SD	3.36 ± 0.34	2.51–4.21	3.81 ± 0.15	3.43–4.18	73.1 ± 1.1	70.5–75.7	50.7 ± 1.9	45.9–55.6
8 SD	4.90 ± 0.75	3.05–6.75	4.81 ± 1.23	1.76–7.87	69.4 ± 2.1	64.1–74.5	39.8 ± 2.3	33.9–45.7

**Table 8:** Pairwise comparison of aerodynamic parameters between formulation pairs. *p*-values from one-way ANOVA with multiple comparison test are reported for each parameter: MMAD, FPF% and RF%. Statistical significance is indicated as follows: *p* < 0.05 (*), *p* < 0.01 (**), *p* < 0.001 (***), ns = not significant.

	MMAD (μm)		FPF%		RF%	
Pair	*p*-Value	Significance	*p*-Value	Significance	*p*-Value	Significance
1 SD vs. 5 SD	0.0369	*	0.0067	**	0.0001	***
2 SD vs. 6 SD	0.0024	**	0.0034	**	<0.0001	***
3 SD vs. 7 SD	0.9864	ns	0.9955	ns	0.8646	ns
4 SD vs. 8 SD	0.3477	ns	0.6276	ns	0.4328	ns

**Table 9:** Z-average (d.nm), diameter (nm) and intensity % of both main and secondary peaks of formulations 1–8 SD, neat serum and purified serum. Results are reported as average values ± standard deviation for dynamic light scattering analysis (n = 3).

		Main Peak		Secondary Peak	
Sample	Z-Average (d.nm)	Size (nm)	Intensity (%)	Size (nm)	Intensity (%)
Neat serum	221.67 ± 13.03	381.17 ± 29.94	79.13 ± 5.20	82.79 ± 18.57	18.43 ± 5.50
F1 SD	108.27 ± 6.29	190.10 ± 9.41	78.70 ± 11.82	31.60 ± 18.65	17.97 ± 13.60
F2 SD	85.45 ± 1.51	189.77 ± 62.04	86.03 ± 6.56	22.90 ± 9.54	13.00 ± 6.68
F3 SD	115.03 ± 2.45	105.25 ± 37.33	55.70 ± 9.86	22.35 ± 8.46	11.83 ± 6.39
F4 SD	144.13 ± 3.47	204.73 ± 8.21	89.73 ± 2.26	30.58 ± 8.08	5.67 ± 0.64
Purified serum	242.97 ± 4.94	388.07 ± 24.45	85.97 ± 2.84	61.54 ± 9.06	13.23 ± 1.62
F5 SD	126.87 ± 4.96	281.00 ± 65.11	82.80 ± 20.33	52.22 ± 48.27	20.70 ± 20.65
F6 SD	112.87 ± 0.91	194.87 ± 15.21	89.43 ± 7.92	22.76 ± 16.67	8.30 ± 7.80
F7 SD	103.83 ± 0.60	199.87 ± 46.30	82.97 ± 20.35	38.32 ± 38.17	20.75 ± 24.11
F8 SD	154.97 ± 3.03	234.07 ± 17.18	94.20 ± 3.86	30.32 ± 13.85	7.25 ± 2.76

**Table 10:** AUC, residual activity and pulmonary activity for the formulations 1–8 SD (mean ± standard deviation, n = 4).

Formulation Nr.	AUC	Residual Activity %	Pulmonary Activity %
1 SD	8009 ± 329	87.9 ± 3.6	68.0 ± 2.6
2 SD	8438 ± 173	92.6 ± 1.9	63.9 ± 1.4
3 SD	8364 ± 220	91.8 ± 2.4	67.8 ± 1.8
4 SD	8250 ± 412	90.6 ± 4.5	64.5 ± 3.1
5 SD	5879 ± 283	82.0 ± 4.0	59.6 ± 2.8
6 SD	5927 ± 484	82.6 ± 6.7	61.1 ± 4.6
7 SD	5444 ± 219	75.9 ± 3.1	55.5 ± 2.1
8 SD	5882 ± 473	82.0 ± 6.6	56.9 ± 4.4

## Data Availability

Data are available upon request to the corresponding author.

## Supplementary Materials

The following supporting information can be downloaded at https://www.mdpi.com/article/10.3390/pharmaceutics17121518/s1: Figure S1: Anti-Spike protein ELISA results of serum and purified serum-based powders at increasing concentration of trehalose. Figure S2: Weight loss as a function of temperature for formulations 1–8 SD, measured by thermogravimetric analysis (TGA).
