# Supplementary material for: Spray-Dried Serum for Inhaled Antiviral Therapy
Source: Pharmaceutics. 2025 Nov 26;17(12):1518. doi: 10.3390/pharmaceutics17121518 (PMC12736332; doi:10.3390/pharmaceutics17121518)
Supplement: Supplementary file 1 [file pharmaceutics-17-01518-s001.zip › Supplementary material/Supplementary Materials Figure S1.docx]

***Supplementary Materials Figure S1***

A

B

C

D


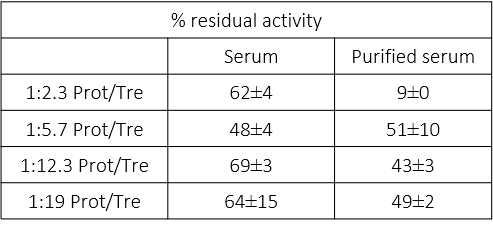


**Figure S1**. Anti-Spike protein ELISA results of serum and purified serum-based powders at increasing concentration of trehalose.

A) Curve of absorbance as a function of proteins’ concentration in a concentration range of 25 – 0.098 μg/mL for spray dried serum-based powders (n=2). Neat serum was used as a reference. B) Curve of absorbance as a function of protein concentration in a concentration range of 25 – 0.098 μg/ml for spray dried serum-based powders (n=2). Neat purified serum was used as a reference. C) Average AUC (n=2) measured for each powder at a protein concentration range of 6.25-0.098 μg/ml compared with respective references. D) Table of the residual activity of IgG in spray-dried powders, calculated as a percentage of the AUC for not spray-dried references.
