# Supplementary material for: Spray-Dried Serum for Inhaled Antiviral Therapy
Source: Pharmaceutics. 2025 Nov 26;17(12):1518. doi: 10.3390/pharmaceutics17121518 (PMC12736332; doi:10.3390/pharmaceutics17121518)
Supplement: Supplementary file 1 [file pharmaceutics-17-01518-s001.zip › Supplementary material/Supplementary Materials Figure S2.docx]

**Supplementary Materials Figure S2**

A

B


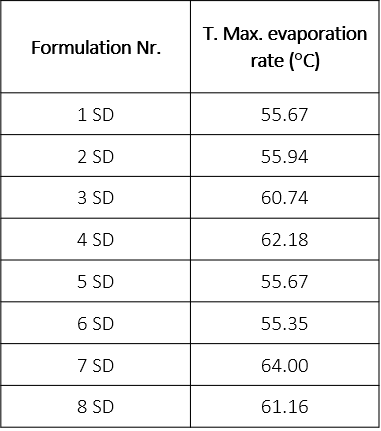


**Figure S2**. (A) Weight loss (% w/w) as a function of temperature for formulations 1–8 SD, measured by thermogravimetric analysis (TGA). Dashed lines indicate the temperature range (55–64 °C) corresponding to the maximum evaporation rate. (B) Table reporting the temperatures at which the maximum evaporation rate occurs, corresponding to the inflection point of the TGA curve for formulations 1–8 SD.
